# N-acetyl-L-cysteine treatment reduces beta-cell oxidative stress and pancreatic stellate cell activity in a high fat diet-induced diabetic mouse model

**DOI:** 10.3389/fendo.2022.938680

**Published:** 2022-08-25

**Authors:** Meg Schuurman, Madison Wallace, Gurleen Sahi, Malina Barillaro, Siyi Zhang, Mushfiqur Rahman, Cynthia Sawyez, Nica Borradaile, Rennian Wang

**Affiliations:** ^1^ Children’s Health Research Institute, London, ON, Canada; ^2^ Department of Physiology & Pharmacology, University of Western Ontario, London, ON, Canada; ^3^ Department of Pathology and Laboratory Medicine, University of Western Ontario, London, ON, Canada

**Keywords:** N-acetyl-L-cysteine (NAC), HFD-induced diabetes, beta-cell oxidative stress, pancreatic stellate cells (PaSCs), collagen fiber

## Abstract

Obesity plays a major role in type II diabetes (T2DM) progression because it applies metabolic and oxidative stress resulting in dysfunctional beta-cells and activation of intra-islet pancreatic stellate cells (PaSCs) which cause islet fibrosis. Administration of antioxidant N-acetyl-L-cysteine (NAC) *in vivo* improves metabolic outcomes in diet-induced obese diabetic mice, and *in vitro* inhibits PaSCs activation. However, the effects of NAC on diabetic islets *in vivo* are unknown. This study examined if dosage and length of NAC treatment in HFD-induced diabetic mice effect metabolic outcomes associated with maintaining healthy beta-cells and quiescent PaSCs, *in vivo*. Male C57BL/6N mice were fed normal chow (ND) or high-fat (HFD) diet up to 30 weeks. NAC was administered in drinking water to HFD mice in preventative treatment (HFD^pNAC^) for 23 weeks or intervention treatment for 10 (HFD^iNAC^) or 18 (HFD^iNAC+)^ weeks, respectively. HFD^pNAC^ and HFD^iNAC+^, but not HFD^iNAC^, mice showed significantly improved glucose tolerance and insulin sensitivity. Hyperinsulinemia led by beta-cell overcompensation in HFD mice was significantly rescued in NAC treated mice. A reduction of beta-cell nuclear Pdx-1 localization in HFD mice was significantly improved in NAC treated islets along with significantly reduced beta-cell oxidative stress. HFD-induced intra-islet PaSCs activation, labeled by αSMA, was significantly diminished in NAC treated mice along with lesser intra-islet collagen deposition. This study determined that efficiency of NAC treatment is beneficial at maintaining healthy beta-cells and quiescent intra-islet PaSCs in HFD-induced obese T2DM mouse model. These findings highlight an adjuvant therapeutic potential in NAC for controlling T2DM progression in humans.

## Introduction

Type 2 diabetes mellitus (T2DM) is a progressive metabolic disease consisting of whole body and pancreatic islet alterations which culminate in dysregulated glycemia (1). T2DM progression, often facilitated by obesity, typically begins with insulin resistance, where peripheral tissue insulin receptors become insensitive and require increased insulin to initiate a response (2, 3). Subclinical inflammation—elevated pro-inflammatory cytokines— is present during obesity which has been associated with metabolic syndrome and peripheral insulin resistance (4). Initially, pancreatic beta-cells accommodate this increase in demand by increasing insulin production as well as their size and number (1, 5). However, this compensatory mechanism is limited and leads to eventual beta-cell failure. The major cause of the clinical progression of T2DM is a decline in beta-cell mass and function within the pancreatic islets (1, 6). Since islet cellular organization is supported by non-islet cells (i.e., pancreatic stellate cells – PaSCs) and the extracellular matrix; this unique microenvironment is crucial for islet function and survival (7–10).

One of the mechanisms facilitating beta-cell failure is believed to be an imbalance between reactive oxygen species (ROS) and antioxidants leading to increased oxidative stress (11). ROS are by-products of metabolism from mitochondria in response to increased glucose, thus in overnutrition settings (i.e. obesity) increased oxidative stress is observed (12). T2DM patients have increased oxidative stress levels compared to non-diabetic controls (11). In addition, diabetic pancreatic islets display lower antioxidant enzyme (i.e. glutathione [GSH], superoxide dismutase [SOD]) expression compared to controls, this further augments beta-cell susceptibility to oxidative stress-induced damage (13, 14). High levels of oxidative stress progresses beta-cell compensation towards dysfunction indicated by reduced nuclear pancreatic and duodenal homeobox 1 (Pdx-1) localization (15). In addition to the beta-cell-specific changes, prolonged high fat diet (HFD)-induced compensation affects the islet microenvironment by increasing intra-islet PaSCs activation and collagen deposition (16). Under conditions of ROS accumulation, mechanical stress and cytokine stimulation, PaSCs are activated and develop a myofibroblast phenotype, gain expression of alpha-smooth muscle actin (aSMA) and are associated with excessive extracellular matrix (ECM) and cytokines production leading to pancreatic inflammation and fibrosis (17, 18). Activated PaSCs are also associated with impaired beta-cell function, *in vitro* (19). However, whether a definitive relationship exists between activated PaSCs and beta-cell dysfunction has not yet been established. Furthermore, it remains to be questioned whether inhibition of PaSC activation and beta-cell oxidative stress *via* antioxidants is a viable therapeutic target for T2DM progression.

N-acetyl-L-cysteine (NAC), a biosynthetic precursor to GSH, is a well-known antioxidant that has been studied for its effect on numerous physiological processes and diseases including T2DM (20–22). In addition to acting as a precursor to glutathione, NAC contains a thiol group which enables it to act directly as an antioxidant; it also acts as an anti-inflammatory (23, 24). NAC has been shown to reduce levels of oxidative stress and improve insulin release in isolated Wistar rat islets (25). When administered in drinking water to HFD murine models of T2DM, NAC improved glucose and insulin tolerance tests (22) and provided functional protection in beta-cells of female mice (26). However, NAC has been shown to be ineffective in limited T2DM clinical work (27). NAC efficacy is impacted by timing (22) and dosage (21) of administration. It was observed to be most beneficial for improving metabolic outcomes when administered as a long-term interventional lasting 5 months (21). Although clinical research is limited, animal models show promise for NAC as a potential treatment in T2DM (21, 22, 26, 28). However, the reported literature lacks understanding with the changes in pancreatic islets particularly in beta-cells and PaSCs following NAC treatment *in vivo*.

In the present study, we aimed to determine the effects of both dosage and timing of NAC administration on pancreatic beta-cells and islet PaSCs in a HFD-induced diabetic mouse model. We hypothesized that the efficiency of NAC treatment will improve metabolic outcomes and beta-cell function by rescuing beta-cell overcompensation while reducing beta-cell stress and PaSC activation induced by HFD. Here, we show that NAC treatment is beneficial for improving metabolic outcomes and beta-cell function, particularly by preserving beta-cell identity, and reducing beta-cell oxidative stress and PaSC activation. However, efficiency of NAC treatment is dependent on both timing and dosage.

## Materials and methods

### Mouse model of high-fat diet induced obesity T2DM with NAC prevention and intervention treatment

Male C57BL/6N (B6N) mice purchased from Charles River (Charles River Laboratories Quebec, Canada) were housed in our facility under a 12:12 h light/dark cycle with a maximum of 5 mice per cage. At 6-weeks of age, mice maintained a normal chow diet (ND) composed of 22% kcal from fat, 23% kcal protein, and 55% kcal from carbohydrates (Harlan Tekard, Indianapolis, IN, USA) or received a high-fat diet (HFD) composed of 60% kcal from fat, 20% from protein, and 20% from carbohydrates (Research Diets INC, New Brunswick, NJ, USA) ad libitum. Mice received a ND or HFD for 22- or 30-week feeding periods (**Supplementary Figure S1**).

Antioxidant treatment with N-acetyl-L-cysteine (NAC) (Santa Cruz Biotechnology, Inc., Santa Cruz, CA, USA) was administered in drinking water to HFD fed mice either in 10mM or 50mM dosage (21, 22). Since the effectiveness of 10mM dose did not show improvement of glucose metabolism (**Supplementary Figure S2**) compared to 50mM dosage, this report was focused on using 50mM NAC for antioxidant groups in a prevention (pNAC) or intervention (iNAC) treatment (**Supplementary Figure S1**). For pNAC treatment, mice received NAC one week prior to HFD and treated through 22-week HFD feeding (HFD^pNAC^). For iNAC treatment, mice received NAC at week 12 after HFD and continued NAC intake for the duration of the 22-week HFD (HFD^iNAC^) and 30-week HFD (HFD^iNAC+^). NAC intake was assessed in each antioxidant treated group (over a three-day period) by measuring water consumption, expressed as NAC intake (mg) per mouse per day. Control mice were age-matched and consumed either ND or HFD with no NAC treatment.

Body weight was monitored weekly, and food intake was measured at the end of the assigned diet period (29). Food intake is expressed kilocalories per mouse per day. All animal work was conducted based on approved protocols from the University of Western Ontario Animal User Subcommittee (Animal Use Protocol #2021-139) in accordance with the Canadian Council of Animal Care guidelines.

### Metabolic studies in experimental mouse models

At the last week of the assigned diet period, fasting blood glucose was examined followed by an intraperitoneal (i.p.) glucose (IPGTT) or insulin (IPITT) tolerance tests. Tests were conducted in the final 2 weeks of diet intervention and at least 5 days apart from one another. For IPGTT, glucose (Dextrose, Sigma, Saint Louis, MO, USA) was administrated at a dose of 2mg/g body weight after an overnight fast for 22 week feeding mice and a 4 hour fast for 30 week feeding mice. For IPITT, insulin (Humulin, Eli Lilly, Toronto, ON, Canada) was administrated at a dose of 1 U/kg body weight after a 4 hour fast. Blood glucose levels were measured before i.p. injection (0 min) and post-injection (15, 30, 60, and 120 mins) and area under the curve (AUC) was used to measure responsiveness. Results of IPITT were normalized to baseline glucose levels (100%) and AUC values were generated to determine changes in insulin sensitivity (30). To determine *in vivo* glucose-stimulated insulin secretion (GSIS), blood samples were collected following 16 h fasting (0 min) and at 5 and 35 min after i.p. injection of glucose (2mg/g). Plasma insulin levels from GSIS and fed cardiac blood was measured using an ultrasensitive insulin mouse ELISA kit (ALPCO, Salem, NH, USA). Plasma triglycerides and cholesterol were measured using enzymatic, colorimetric assays (Wako Diagnostics, Mountain View, California, USA).

### Immunohistological staining and morphometric analyses

At the end of experimental time points, pancreases from mice were collected, weighed, and fixed in 4% paraformaldehyde. Whole pancreatic tissues were embedded in paraffin and sectioned consecutively at 4-µm throughout the length of the pancreas, from head to tail, to avoid any bias due to regional changes in islet distribution and islet cell composition of the pancreas (31). Sections were incubated with the appropriate dilutions of primary antibodies as listed in **Supplementary Table S1**. As required, 0.2% Triton treatment and/or microwave antigen retrieval with citric acid solution (pH 6.0) were applied to improve detection. Secondary antibodies consisted of fluorescein isothiocyanate (FITC) and tetramethyl rhodamine isothiocyanate (TRITC) (1:50; Jackson Immunoresearch, West Grove, PA, USA). 4’-6’-diamidino-2-phenylindole (DAPI) was used for nuclear counterstaining (Sigma-Aldrich). Images were captured using Nikon Eclipse Ti2 Confocal Microscope (Nikon, Melville, NY, USA) followed by morphometric analysis of islet density, alpha and beta-cell mass and beta-cell size, as previously described (30, 32). Beta-cell proliferation, expression of Pdx-1 transcription factor and oxidative stress were identified by double immunofluorescence staining and quantification from at least 10 random islets per pancreas section. Double positive cells (nuclear marker-positive with insulin-positive) were divided by the total insulin-positive cell population in islets and expressed as a percentage (33). To quantify the percentage of intra-islet PaSCs population, labeled by αSMA and desmin, the positive intra-islet αSMA^+^ or desmin^+^ cell area (µm^2^) was manually traced using Nikon NIS-Element software and divided by the total insulin-positive area (µm^2^).

Trichrome staining kit (Abcam, Cambridge, MA, USA) was used to measure intra-islet collagen deposition (34). Stained images were acquired at 40x magnification using an Aperio AT2 whole slide scanner (Leica Biosystems Inc, Concord, Ontario, Canada). The percentage of islet collagen deposition was determined using an ImageJ macro, which segments blue areas (collagen) from red areas in the image using colour deconvolution and measures total blue area per image as previously described (35).

### Statistical analysis

All data are expressed as means ± SEM. Statistical significance was analyzed using GraphPad Prism (version 6 GraphPad Software, San Diego, CA, USA) with 95% of confidence interval. The difference was analyzed using one-way ANOVA followed by Tukey’s *Post-Hoc* test. Differences were considered to be statistically significant when p <0.05.

## Results

### NAC treatment improves glucose metabolism as dose- and time-dependent in HFD-induced diabetic mice

To determine the efficiency of NAC treatment in HFD-induced diabetic mice, both dose- and time-dependent study was established. Dosages of 10mM and 50mM NAC treatment were administered for prevention and intervention groups. HFD mice with 10mM NAC preventive or interventive treatment did not show changes in body weight or fasting blood glucose and had no improvement of glucose metabolism as determined by glucose and insulin tolerance tests (**Supplementary Figure S2**). Therefore, this report only focused on 50mM dosage of NAC treatment.

**Figure 1:**
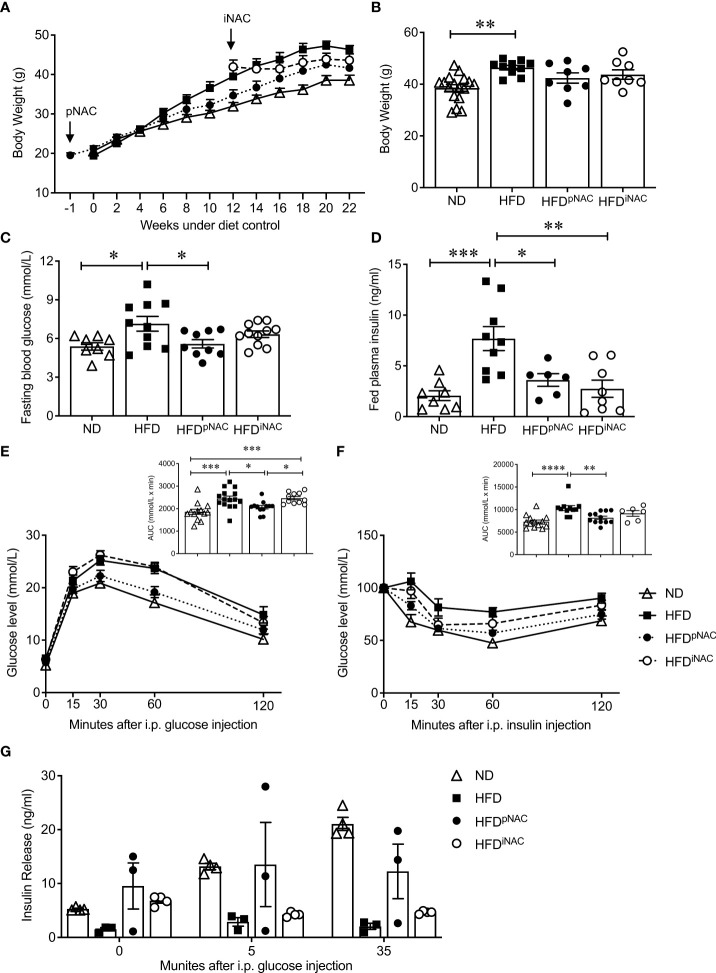
Preventive NAC treatment improves glucose metabolism in HFD-induced diabetic mice. **(A)** Recording of bi-weekly body weight for the duration of 22 weeks (n=8-17 mice/group). Measurement of **(B)** body weights, **(C)** overnight (~16 hours) fasting blood glucose and **(D)** fed plasma insulin levels (n=6-17 mice/group). **(E)** IPGTT and area under curve (AUC, n=10-14 mice/group), **(F)** IPITT and AUC (n=6-12 mice/group) and **(G)** *in vivo* GSIS (n=3-5 mice/group) of ND, HFD, HFD^pNAC^ and HFD^iNAC^ mice at 22 weeks. Control diets (ND): open triangle; HFD: closed square; HFD^pNAC^: closed circle; HFD^iNAC^: open circle. Data are expressed as means ± SEM. *p<0.05, **p<0.01, ***p<0.001, ****p<0.0001; analyzed using one-way ANOVA followed by Tukey’s *Post-Hoc* test.

Bi-weekly body weights were recorded for the duration of treatment (**Figure 1A**). HFD mice showed significantly increased body weight compared to ND. There were no body weight significant differences between HFD, HFD^pNAC^ and HFD^iNAC^ groups (**Figure 1B**). Significant differences in average NAC intake were noted with highest NAC intake observed in the HFD^pNAC^ group (21mg/mouse/day) compared to HFD^iNAC^ (14mg/mouse/day) group and HFD^iNAC+^ (16mg/mouse/day) group (**Supplementary Figure S3A**). Only HFD^iNAC^ group showed significantly increased food intake when compared to ND group (**Supplementary Figure S3B**). A significantly increased fasting blood glucose was determined in the HFD mice when compared to ND (**Figure 1C**), however, an improved fasting blood glucose level was only observed in HFD^pNAC^, not HFD^iNAC^ mice (**Figure 1C**). Fed plasma insulin level was found to be significantly elevated in HFD mice compared to all other groups, and both HFD^pNAC^ and HFD^iNAC^ mice displayed a similar plasma insulin level as ND mice (**Figure 1D**). Fed circulating triglycerides and cholesterol were assessed and showed no significant differences between ND, HFD, and NAC treated HFD groups (**Supplementary Figure S4**).

Mice under HFD for 22 weeks demonstrated a progressive T2DM-like phenotype with impaired glucose and insulin tolerance when compared to ND mice (**Figures 1E, F** **)**. Significantly improved glucose tolerance and insulin sensitivity in HFD^pNAC^ mice was determined, which showed similar response as ND mice, however, this improvement was not found in HFD^iNAC^ mice (**Figures 1E, F** **)**. Furthermore, *in vivo* GSIS showed an observable improvement of glucose stimulated insulin secretion in HFD^pNAC^ mice compared to HFD and HFD^iNAC^ groups (**Figure 1G**). These data indicate that timing efficiency of NAC treatment is critical to normalize glucose metabolism in HFD-induced diabetic mice.

### NAC treatment preserves beta-cell mass and identity with reduction of beta-cell oxidative stress in HFD-induced diabetic mice

**Figure 2:**
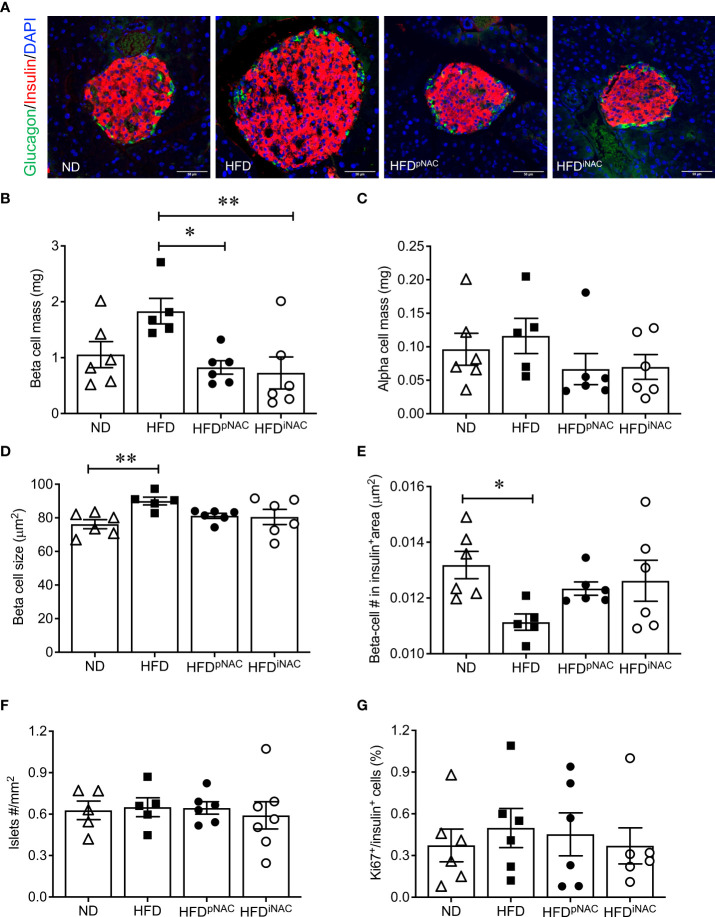
Histological analysis of NAC treated HFD pancreata demonstrated preserved beta-cell mass with no change in cell proliferation. **(A)** Representative double immunofluorescence images for islet morphology, detected by insulin (red) and glucagon (green) staining, and DAPI labeled nuclei (blue). Scale bars: 50µm. **(B)** Beta cell mass **(C)** alpha cell mass **(D)** beta cell size and **(E)** number **(F)** islet density in ND, HFD, HFD^pNAC^ and HFD^iNAC^ mouse pancrea at 22 weeks (n=5-6 pancreata/group). **(G)** Proliferation of beta cells quantified using Ki67 co-localization with insulin^+^ cells (n=6 pancreata/group). ND: open triangle; HFD: closed square. HFD^pNAC^: closed circle; HFD^iNAC^: open circle. Data are expressed as means ± SEM. *p<0.05, **p<0.01, analyzed using one-way ANOVA followed by Tukey’s *Post-Hoc* test.

Double immunofluorescence staining for insulin and glucagon were used for determining islet density, beta- and alpha-cell mass, and beta-cell size (**Figure 2A** **)**. Morphological analysis of beta-cell mass showed an increase in HFD pancreas, with no discernable changes in alpha cell mass or islet density (**Figures 2B, C, F**). The increased beta-cell mass in HFD mice was due to an enlargement in beta-cell size with low beta-cell number in the insulin positive area (**Figures 2D, E** **)** but was not caused by changes in beta-cell proliferation as determined using Ki67 co-staining (**Figure 2G**). Interestingly, HFD-induced enlargement of beta-cell mass was not observed in HFD^pNAC^ and HFD^iNAC^ groups (**Figure 2B**). Beta-cell mass, size, and number in HFD^pNAC^ and HFD^iNAC^ mouse islets displayed similar results as ND mice (**Figures 2D, E** **)**.

**Figure 3:**
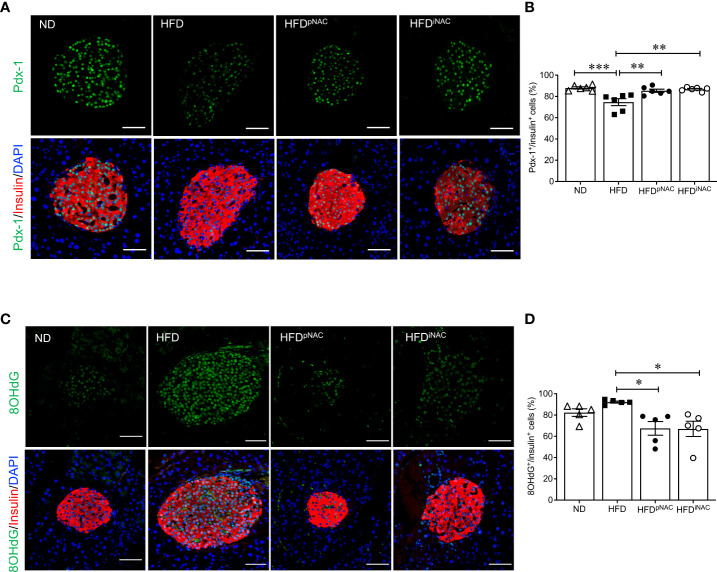
NAC treatment preserves beta-cell identity with reduction of beta-cell oxidative stress in HFD-induced diabetic mice. Representative double immunofluorescence images of **(A)** Pdx-1 (green) **(C)** 8OHdG (green) co-stained with insulin (red), nuclei are stained with DAPI (blue). Scale bars: 50µm. Quantification of nuclear **(B)** Pdx-1 **(D)** 8OHdG in insulin^+^ cells of ND, HFD, HFD^pNAC^ and HFD^iNAC^ mouse pancreas at 22 weeks (n=5-6 pancreata/group). ND: open triangle; HFD: closed square; HFD^pNAC^: closed circle; HFD^iNAC^: open circle. Data are shown as means ± SEM. *p<0.05, **p<0.01, ***p<0.001, analyzed using one-way ANOVA followed by Tukey’s *Post-Hoc* test.

When analyzing transcription factors (Pdx-1, MafA, and FoxO1) involved in beta-cell function and insulin secretion, it was found that nuclear Pdx-1 localization was significantly reduced in HFD mouse beta-cells compared to ND, HFD^pNAC^ and HFD^iNAC^ groups (**Figures 3A, B** **)**. Both HFD^pNAC^ and HFD^iNAC^ groups showed significantly improved Pdx-1 nuclear localization, similar to ND group (**Figures 3A, B** **)**. No obvious changes were found between experimental mice for MafA and FoxO1 nuclear localization in beta-cells (data not shown).

To examine NAC’s impact on beta-cell oxidative stress, presence of 8OHdG in beta-cells was quantified using double staining for 8OHdG and insulin. The percentage of nuclear 8OHdG labelling in insulin-positive cells was significantly higher in the HFD mice than that of HFD^pNAC^ and HFD^iNAC^ groups (**Figures 3C, D** **)**. A significant reduction of beta-cell 8OHdG labelling occurred in both NAC treated HFD mice, but no change observed in the ND mice when compared to HFD mice (**Figures 3C, D** **)**.

### NAC treatment reduces active PaSCs in islets leading to lower intra-islet collagen deposition in HFD-induced diabetic mice

**Figure 4:**
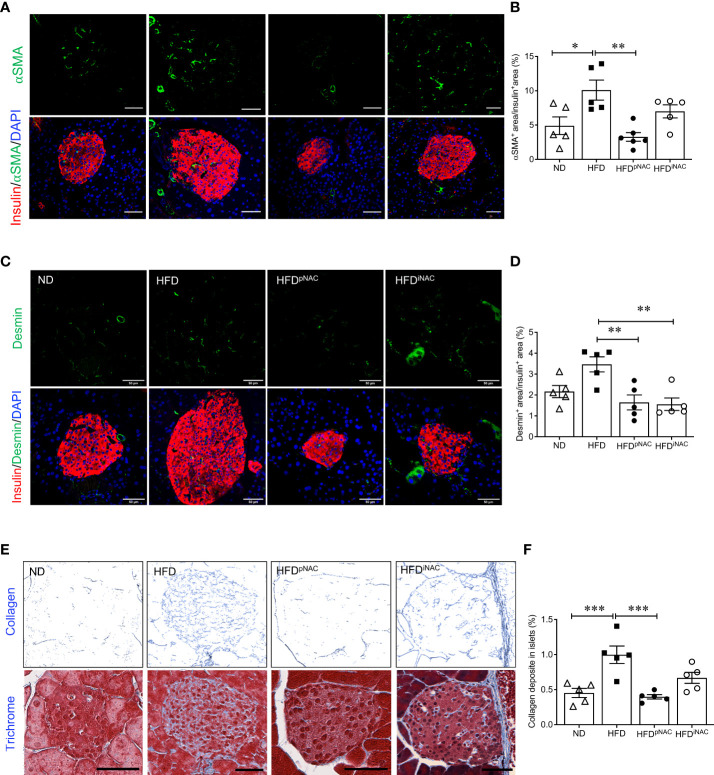
Preventive NAC treatment reduces intra-islet PaSCs activation and collagen deposition in HFD-induced diabetic mice. Representative double immunofluorescence images of **(A)** α-SMA (green) or **(C)** desmin (green) co-stained with insulin (red), nuclei are stained with DAPI (blue). Scale bars: 50µm. Quantification of intra-islet α-SMA **(B)** or desmin **(D)** area in ND, HFD, HFD^pNAC^ and HFD^iNAC^ mouse islets at 22 weeks (n=5-6 pancreata/group). **(E)** Representative trichrome staining images, scale bar: 50μm; and **(F)** quantification of intra-islet collagen deposition (n=5 pancreata/group). ND: open triangle; HFD: closed square; HFD^pNAC^: closed circle; HFD^iNAC^: open circle. Data are shown as means ± SEM. *p<0.05, **p<0.01, ***p<0.001, analyzed using one-way ANOVA followed by Tukey’s *Post-Hoc* test.

To determine whether NAC influenced intra-islet PaSCs activation during HFD induced oxidative stress, quantification of active PaSCs population was performed. Since marker αSMA labels active PaSCs and desmin marks both quiescent and active PaSCs, double immunofluorescence staining for αSMA and desmin was performed to verify their colocalization (**Supplementary Figure S5**). Quantification of intra-islet activated PaSCs labeled by αSMA staining revealed a significant increase in αSMA area in HFD mouse islets compared to ND and HFD^pNAC^ mice (**Figures 4A, B** **)**. Although, αSMA^+^ staining in HFD^iNAC^ islets showed a slight reduction, there was no statistically significant differences between HFD and HFD^iNAC^ mice (**Figures 4A, B** **)**. Measuring desmin population in the experimental groups showed a significantly higher percentage of desmin^+^ staining present in HFD mouse islets compared to HFD^pNAC^ and HFD^iNAC^ mouse islets, but no significance observed in ND group (**Figures 4C, D** **)**. Both data indicate that HFD induced PaSC activation could be diminished by NAC administration.

To further identify whether NAC treatment could abolish HFD-induced PaSC activation and subsequent intra-islet collagen deposition of islet fibrosis, quantification of intra-islet collagen deposition was performed using trichrome stain (**Figure 4E**). Intra-islet collagen deposition was significantly increased in HFD mouse islets compared to ND mouse islets (**Figure 4F**), indicating HFD results in progression of islet fibrosis. A significant reduction of intra-islet collagen deposition in HFD^pNAC^ mouse islets was found (**Figures 4E, F** **)**, but this observation was not present in HFD^iNAC^ mouse islets (**Figures 4E, F** **)**.

### Prolonged intervention NAC treatment improves glucose metabolism in HFD-induced diabetic mice

**Figure 5:**
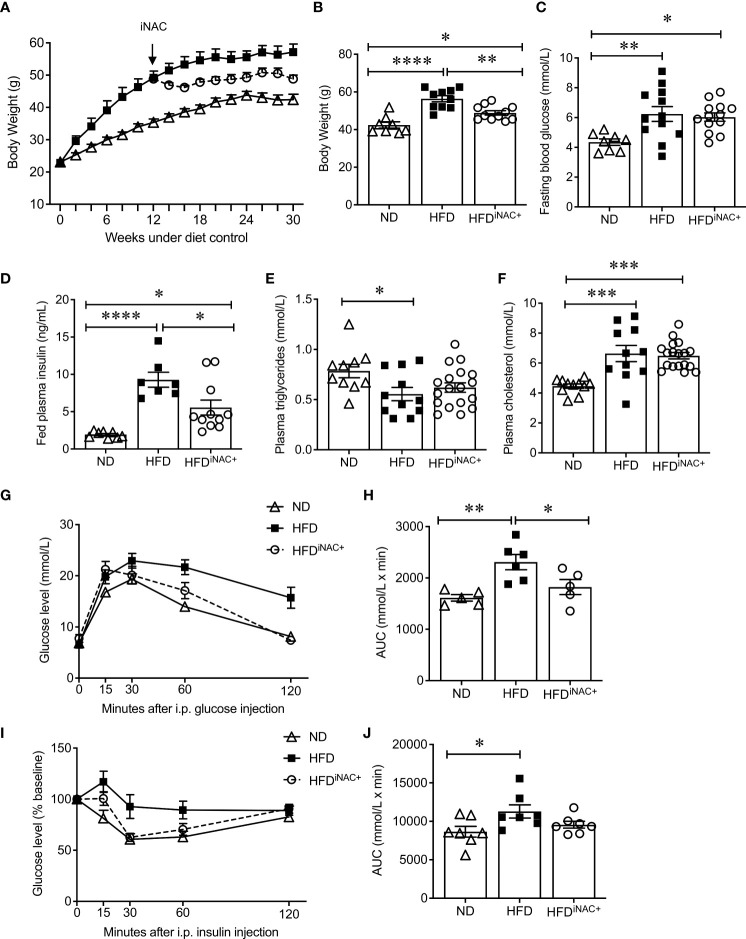
Prolonged intervention NAC treatment improves glucose metabolism in HFD-induced diabetic mice. **(A)** Recording of biweekly body weight for the duration of 30 weeks (n=8-12 mice/group). Measurement of **(B)** body weights, **(C)** overnight (~16 hours) fasting blood glucose, **(D)** fed plasma insulin, **(E)** triglycerides and **(F)** cholesterol levels (n=7-18 mice/group). **(G)** IPGTT and **(H)** AUC (n=5-6 mice/group), **(I)** IPITT and **(J)** AUC (n=7 mice/group) of ND, HFD and HFD^iNAC+^ mice at 30 weeks. ND: open triangle; HFD: closed square; HFD^iNAC+^: open circle. Data are expressed as means ± SEM. *p<0.05, **p<0.01, ***p<0.001, ****p<0.0001, analyzed using one-way ANOVA followed by Tukey’s *Post-Hoc* test.

Compared to HFD^pNAC^ mice, HFD^iNAC^ mice received NAC treatment 12 weeks after HFD initiation with total duration of 10 weeks NAC treatment. HFD^iNAC^ mice showed no improvement in glucose metabolism, PaSCs activation or collagen deposition, indicating the duration of NAC treatment is critical. Therefore, a prolonged NAC treatment of up to 18 weeks (HFD^iNAC+^) was established and investigated. Bi-weekly body weights were recorded up to 30 weeks (**Figure 5A**). HFD-fed mice had significantly increased body weight compared to ND-fed mice and HFD^iNAC+^ mice (**Figure 5B**). Reduced body weight was observed in HFD^iNAC+^ mice compared to HFD mice but remained significantly higher than that of ND mice (**Figure 5B**). Overnight fasting blood glucose was significantly elevated in both HFD and HFD^iNAC+^ mice compared to ND mice (**Figure 5C**). However, hyperinsulinemia observed in HFD mice were significantly improved in HFD^iNAC+^ mice, but remained significantly higher levels compared to ND mice (**Figure 5D**). Fed plasma triglyceride levels were significantly lower in HFD mice, but HFD^iNAC+^ mice showed relatively elevated plasma triglycerides similar to ND mice (**Figure 5E** **)**. Fed plasma cholesterol showed significant increase in both HFD and HFD^iNAC+^ mice when compared to ND mice (**Figure 5F**). A significant impairment of glucose tolerance was present in HFD mice that was not observed in HFD^iNAC+^ mice. HFD^iNAC+^ mice which displayed similar glucose tolerance as ND mice (**Figures 5G, H** **)**. A significant impairment of insulin tolerance was observed in HFD mice when compared to ND mice, however, insulin sensitivity showed a trend toward improvement in HFD^iNAC+^ mice compared to HFD mice, but statistical significance was not reached (**Figures 5I, J** **)**.

### Prolonged intervention NAC treatment preserves beta-cell mass and reduces intra-islet PaSCs activation in HFD-induced diabetic mice

**Figure 6:**
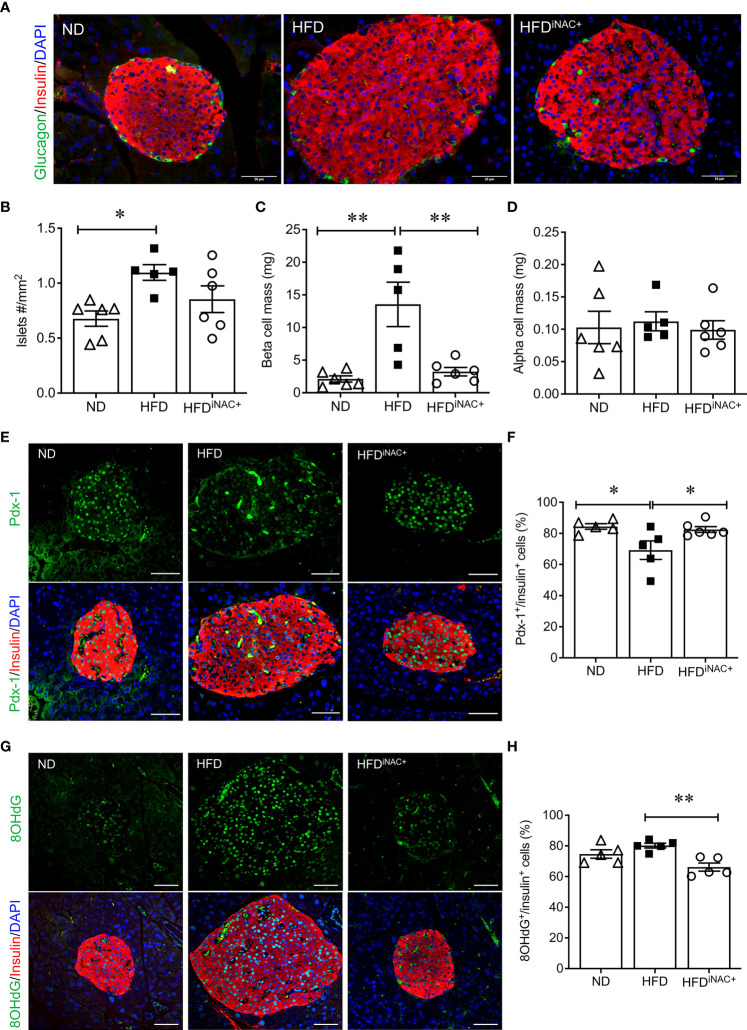
Prolonged intervention NAC treatment preserves beta-cell mass and nuclear Pdx-1 with lower 8OHdG in HFD-induced diabetic mice. **(A)** Representative double immunofluorescence images for islet morphology, detected by insulin (red) and glucagon (green) staining and DAPI labeled nuclei (blue). Scale bars: 50µm. Morphometric quantification of islet density **(B)** beta cell mass **(C)** and alpha cell mass **(D)** (n=5-6 pancreata/group). Representative double immunofluorescence images for **(E)** Pdx-1 (green) or **(G)** 8OHdG (green) and insulin (red), nuclei are stained with DAPI (blue). Scale bars: 50µm. Quantification of nuclear Pdx-1 **(F)** or 8OHdG **(H)** in insulin^+^ cells of ND, HFD and HFD^iNAC+^ mouse pancreas at 30 weeks (n=5-6 pancreata/group). ND: open triangle; HFD: closed square). HFD^iNAC+^: open circle. Data are shown as means ± SEM. *p<0.05, **p<0.01, analyzed using one-way ANOVA followed by Tukey’s *Post-Hoc* test.

**Figure 7:**
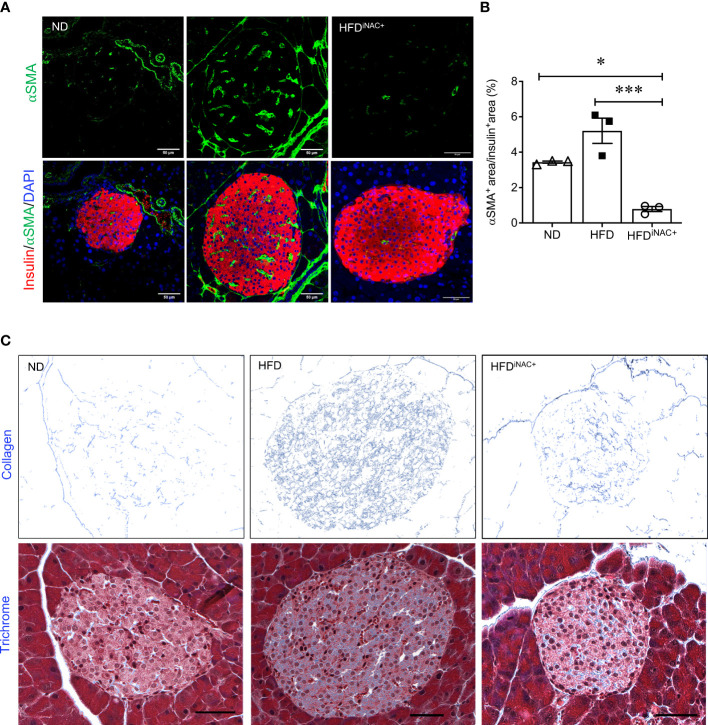
Prolonged intervention NAC treatment reduces intra-islet PaSCs activation and collagen deposition in HFD-induced diabetic mice. **(A)** Representative double immunofluorescence images of α-SMA (green) co-stained with insulin (red), nuclei are stained with DAPI (blue). Scale bars, 50µm; **(B)** quantification of intra-islet α-SMA area in ND, HFD and HFD^iNAC+^ mouse islets at 30 weeks (n=3 pancreata/group). **(C)** Representative trichrome staining images of intra-islet collagen deposition. Scale bar: 50μm. ND: open triangle; HFD: closed square; HFD^iNAC+^: open circle) Data are shown as means ± SEM. *p<0.05, ***p<0.001, analyzed using one-way ANOVA followed by Tukey’s *Post-Hoc* test.

Double immunofluorescence staining images for insulin and glucagon showed large islets present in HFD and HFD^iNAC+^ mouse pancreas compared to ND group (**Figure 6A**). Morphometric analysis of islet number showed HFD mice have significantly elevated islet numbers compared to ND, however, islet number in HFD^iNAC+^ is comparable to ND (**Figure 6B**). Beta-cell mass was significantly increased in HFD and was significantly improved in HFD^iNAC+^ mice; HFD^iNAC+^ mice and ND mice showed similar beta-cell masses (**Figure 6C**). There were no differences observed in alpha-cell mass found between groups (**Figure 6D**). HFD induced beta-cell dysfunction with loss of nuclear Pdx-1 signals was significantly rescued in HFD^iNAC+^ islets, which showed similar nuclear Pdx-1 localization as ND islets (**Figure 6F**). In parallel, HFD induced beta-cell oxidative stress labeled by 8OHdG^+^ was also significantly improved in HFD^iNAC+^ islets (**Figures 6G, H** **)**. Furthermore, HFD^iNAC+^ mice showed significantly lower intra-islet αSMA labeling (**Figures 7A, B** **)** with improved intra-islet collagen deposition (**Figure 7C**), which was not observed in HFD^iNAC^ islets receiving a shorter duration of NAC treatment. HFD mice maintained higher level of αSMA labeling and collagen deposition in the intra-islets when compared to ND and HFD^iNAC+^ mice (**Figure 7**). This data further verified that NAC treatment improves beta-cell identity, oxidative stress and PaSC induced fibrotic in HFD-induced diabetic islets is in a time-dependent manner.

## Discussion

**Figure 8:**
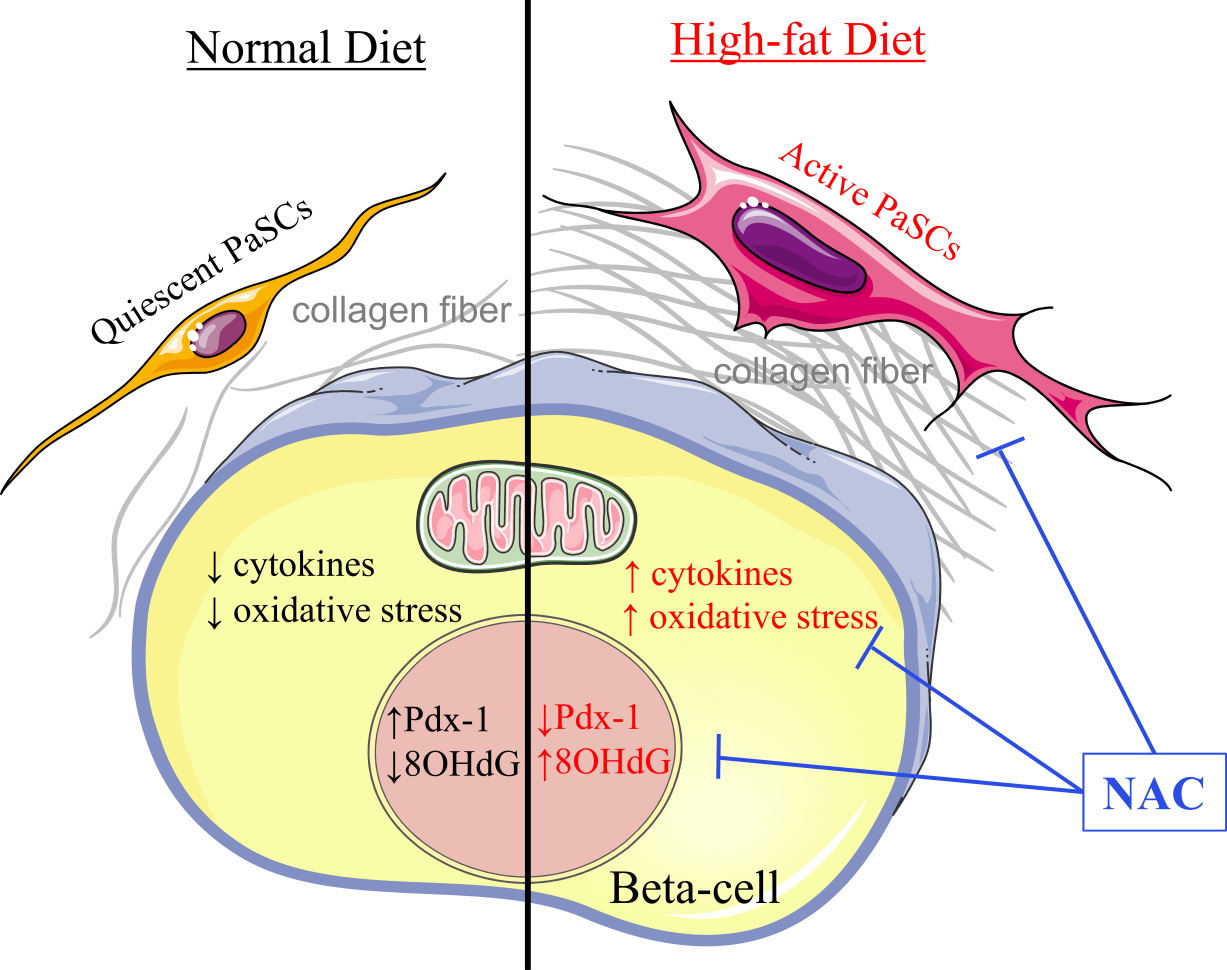
Proposed model of NAC maintains healthy beta-cells and quiescent PaSCs in HFD-induced diabetic mouse islet. In a healthy condition under normal chow diet, intra-islet PaSCs are quiescent; they produce suitable cytokines and ECM for maintaining islet structure and facilitating healthy beta-cell function. Under high-fat diet, increased oxidative stress promoted intra-islet PaSC activation resulting in increased cytokine production and ECM deposition which diminished beta-cell identity and function. Given NAC treatment, HFD islets showed significantly reduced oxidative stress and intra-islet PaSC activation with enhanced beta-cell identity and function.

This is a first *in vivo* study investigating the effects of antioxidant NAC on diabetic islets in HFD-induced diabetic mice. It is well documented that mice fed with HFD display dysregulation of glucose metabolism and beta-cell compensation with increased beta-cell oxidative stress and intra-islet PaSC activation, which produces excessive ECM and cytokines resulting in islet fibrosis (**Figure 8**). When HFD-induced diabetic mice were administered NAC at an effective dose and time, significantly improved glucose tolerance and insulin sensitivity, and rescue from beta-cell overcompensation was determined. NAC treated HFD mice displayed a normalized beta-cell mass and size with significantly improved beta-cell identity, as Pdx-1 nuclear localization, and reduced beta-cell oxidative stress, labeled by 8-OHdG (**Figure 8** **)**. Importantly, NAC diminished intra-islet PaSCs activation and PaSC-induced collagen deposition preventing fibrosis caused by HFD (**Figure 8**). This study suggests that antioxidant therapy using NAC is beneficial for maintaining healthy beta-cells and intra-islet quiescent PaSC population in HFD-induced diabetic mice and provides a better understanding of effective dose and time for antioxidant therapy in obesity related diabetes in humans.

Identifying the optimal dosage during NAC therapy is critical for designing clinical trials. Use of sub-optimal or excessive antioxidant dosages of NAC could hinder NAC’s positive anti-diabetic effects in T2DM patients (24). Based on previous reports (20, 21), two dosages of NAC (10mM and 50mM) were employed in this study and showed HFD mice that received 10mM NAC in drinking water for up to 22 weeks resulted in no improvements in glucose tolerance and insulin sensitivity. Whereas, administration at 50mM in drinking water showed significant improvements in glucose metabolism, which was consistent with the findings by Falach-Malik et al. (21). It was noted that NAC at 50mM dosage did not cause a significant reduction in body weights, despite increased caloric intake in both HFD^pNAC^ and HFD^iNAC^ mice under 22 weeks HFD feeding. Previous reports suggest that this dosage potentially caused an increase in motor activity and/or metabolic activity which may have been compensated for by increasing food intake resulting in no net loss of weight (22, 36). However, prolonged intervention NAC in HFD^iNAC+^ mice displayed significantly reduced body weights compared to HFD mice under 30 weeks HFD feeding, suggesting NAC treatment is associated with weight loss and may account for required time to take effect.

Previous studies suggest using NAC at an earlier time point increased effectiveness for improving glucose tolerance and insulin sensitivity related to weight loss in HFD mice (21, 22). HFD mice that received preventive NAC treatment for 23 weeks showed significantly improved glucose and insulin tolerance, but this was not observed at 10 weeks. Prolonged intervention NAC (18 weeks) administration to HFD mice displayed improvement similar to NAC prevention treated mice, indicating that positive anti-diabetic effects of NAC in HFD-induced diabetic mice require up to 18 weeks of prevention or intervention NAC treatment. Although, there was no change in fed plasma lipid level in mice with 22 weeks HFD feeding, HFD-induced hepatic insufficient export of triglycerides was observed in HFD mice with 30 weeks feeding, this impaired liver export of triglycerides was rescued in HFD^iNAC+^ mice that showed normalised triglycerides level comparable to ND mice. This data indicates that NAC improved glucose metabolism and may be associated with improved liver function to reduce hepatic triglyceride accumulation in order to reduce gluconeogenesis and enhance insulin signalling (37, 38). Taken together, this study suggests that adequate duration of administration is crucial for NAC to be effective as an antioxidative therapy in treating diet-induced obesity diabetes.

Mice fed with HFD for 22 or 30 weeks showed not only impaired metabolic outcomes, but also significant alterations to islet architecture indicating a beta-cell compensation response. HFD mice with NAC prevention or intervention treatment can prevent or rescue this beta-cell overcompensation with significantly normalized beta-cell mass and size. This demonstrates that NAC is effective at maintaining beta-cell function during HFD-induced stress. It is agreed that transcription factor Pdx-1 plays a critical role in regulating beta-cell function by activating genes essential for beta-cell identity; loss of beta-cell identity contributes to the pathogenesis of type 2 diabetes (39). A previous study by Leenders et al. proposed under elevated oxidative stress conditions, beta-cells dedifferentiate into other islet cell types leading to reduced beta-cell identity (40). In the present study, loss of beta-cell identity and increased beta-cell oxidative stress was displayed in HFD mice, however, HFD^pNAC^ and HFD^iNAC^ islets showed preserved or restored beta-cell Pdx-1 nuclear localization, and reduced beta-cell oxidative stress. These findings are supported by previous reports that demonstrated NACs ability to improve Pdx-1 and insulin content in beta-cells in a genetic obesity model of T2DM (26), and to prevent glucose toxicity-induced impaired Pdx-1 binding to the insulin gene promoter in beta-cell line *in vitro* and reduce plasma 8-OHdG oxidative stress level in Zucker diabetic fatty rats (41). Although the exact mechanism of NACs action on beta-cells remains to be elucidated, it is a well-known that NAC is a biosynthetic precursor of glutathione (23). It also contains a thiol group exerting its antioxidant effects through electron donation (42) and has recently been investigated for its potential intracellular conversion to sulfane sulfur species to scavenge oxidants (43). NAC also produces anti-inflammatory actions by indirectly inhibiting the activation of the redox-sensitive pathway NF-κB in order to prevent inflammatory responses which lead to loss of Pdx-1 expression (42). Since the present study did not evaluate inflammatory markers, it would be interesting for future studies to investigate the role of NAC on inflammatory markers in the setting of diet-induced obesity.

PaSCs in islets play a critical role in controlling ECM, growth factors and cytokine secretion. Over-active PaSCs are involved in beta-cell compensation in the early stages of T2DM progression (44). Furthermore, clinical studies have shown higher levels of αSMA and islet fibrosis in T2DM patients (45), such evidence was also observed in the current study where HFD mice showed significantly elevated intra-islet αSMA^+^ cells with excessive collagen deposition. Administration of NAC to HFD mice displayed significantly reduced intra-islet oxidative stress and intra-islet αSMA^+^, which corresponded to significantly reduced intra-islet fibrosis. This observation matches previous *in vitro* reports that NAC is capable of reducing PaSC activation and reverting cells to a quiescent state (46–48). NAC treatment results in increased vitamin A droplets indicative of quiescent PaSCs (47). It was noted that prevention NAC treated HFD^pNAC^ mice showed similar PaSC activation and islet collagen staining as ND islets, suggesting that initiating NAC therapy is most beneficial to prevent PaSCs activation and intra-islet collagen deposition caused by HFD. However, intervention NAC treated islets did not show a significant improvement until 18 weeks NAC treatment, indicating PaSCs activation begin rapidly after HFD-induced stress, and reverting active PaSCs and collagen accumulation requires sufficient time with antioxidant treatment. A study on diabetic cardiac function in C57/B6 mice supports these results as they determined NAC is beneficial at improving cardiac function and reducing cardiac fibrosis, most noticeably in mice which received earlier and longer treatment (28). However, the potential mechanisms of NAC action on intra-islet PaSCs require further *in vivo* investigation.

In summary, the present study provides evidence that NAC is effective at protecting beta-cells from overcompensation and loss of beta-cell identity observed during T2DM progression in mice. The efficacy of NAC treatment in HFD-induced diabetic mice was dependent on dosage and duration of treatment. This study supports previous reports showing the ability of NAC to improve metabolic outcomes in mouse models of T2DM. Importantly, it provides first evidence of NAC’s effect on the intra-islet environment where beta-cell identity was protected, and oxidative stress and PaSC activation was diminished. Ultimately, the demonstrated benefits of antioxidant NAC on diet-induced obesity and diabetes indicates that it should be further investigated as an adjuvant therapy in controlling T2DM progression in humans.

## Data availability statement

The original contributions presented in the study are included in the article/**Supplementary Material**. Further inquiries can be directed to the corresponding author.

## Ethics statement

The animal study was reviewed and approved by University of Western Ontario Animal User Subcommittee.

## Author contributions

MS and MW contributed to an *in vivo* mouse feeding and glucose metabolic study, data acquisition, interpretation of data, and preparation of the submitted manuscript. MB contributed to the maintenance of animals, data acquisition and preparation of the submitted manuscript. GS, MR, SZ and CS contributed to data acquisition. NB contributed interpretation of data and manuscript discussion. RW contributed to the design of experiments within this project, interpretation of data and preparation of the submitted manuscript. All authors contributed to the article and approved the submitted version.

## Funding

This study was funded by the Canadian Institutes of Health Research (grant# 152944).

## Acknowledgments

This work was supported by grants from the Canadian Institutes of Health Research.

## Conflict of interest

The authors declare that the research was conducted in the absence of any commercial or financial relationships that could be construed as a potential conflict of interest.

## Publisher’s note

All claims expressed in this article are solely those of the authors and do not necessarily represent those of their affiliated organizations, or those of the publisher, the editors and the reviewers. Any product that may be evaluated in this article, or claim that may be made by its manufacturer, is not guaranteed or endorsed by the publisher.

## Supplementary material

Supplementary Figure 1 Experimental timeline to determine the time-dependent of NAC administration on high fat diet-induced diabetic mice. Both normal diet (ND) and high fat diet (HFD) experimental groups were established at age of 6 weeks. pNAC: preventative NAC was started at 5 weeks of age, one week prior to HFD diet start and had a total of 23 weeks of NAC treatment; iNAC: intervention NAC was given at 18 weeks of age, 12 weeks after HFD diet start and had a total of 10 weeks of NAC treatment; iNAC^+^: intervention NAC was given at 18 weeks of age, 12 weeks after HFD diet start and had a total of 18 weeks of NAC treatment.

Supplementary Figure 2 10mM NAC treatment did not improve glucose metabolism in HFD-induced diabetic mice. **(A)** Body weight, **(B)** fasted blood glucose levels, **(C)** IPGTT, **(D)** area under curve (AUC) analysis of IPGTT, **(E)** IPITT and **(F)** AUC analysis of IPITT results from ND, HFD, HFD^pNAC^ and HFD^iNAC^ mice during 22-week experiment. Data are expressed means ± SEM (n=3-17 mice per experimental group). *p<0.05, **p<0.01, ***p<0.001 vs. ND, determined by one-way ANOVA followed by Tukey’s multiple comparisons test.

Supplementary Figure 3 NAC and food intake in HFD-induced diabetic mice. **(A)** Average NAC intake for mice undergoing prevention (pNAC), intervention (iNAC) and extend intervention (iNAC+) treatment. **(B)** Food intake in ND, HFD, HFD^pNAC^ and HFD^iNAC^ mice and **(C)** Food intake in ND, HFD and HFD^iNAC+^ mice. Data are expressed means ± SEM (*n=5-11* mice per experimental group). *p<0.05, **p<0.01, ***p<0.001 vs. ND or as indicated, determined by one-way ANOVA followed by Tukey’s multiple comparisons test.

Supplementary Figure 4 Plasma lipid levels in ND, HFD, HFD^pNAC^ and HFD^iNAC^ mice. Fed plasma triglycerides **(A)** and cholesterol **(B)**. Data are expressed means ± SEM (*n=8-11* mice per experimental group).

Supplementary Figure 5 Correlation of desmin (green) and αSMA (red) in the islet as dash-line outlined. Lower panel displays enlarged images, arrows indicate co-staining, arrowheads label single staining. Scale bar: 50μm.
