# Supplementary figures and images for: N-acetyl-L-cysteine treatment reduces beta-cell oxidative stress and pancreatic stellate cell activity in a high fat diet-induced diabetic mouse model
Source: Front Endocrinol (Lausanne). 2022 Aug 25;13:938680. doi: 10.3389/fendo.2022.938680 (PMC9452715; doi:10.3389/fendo.2022.938680)

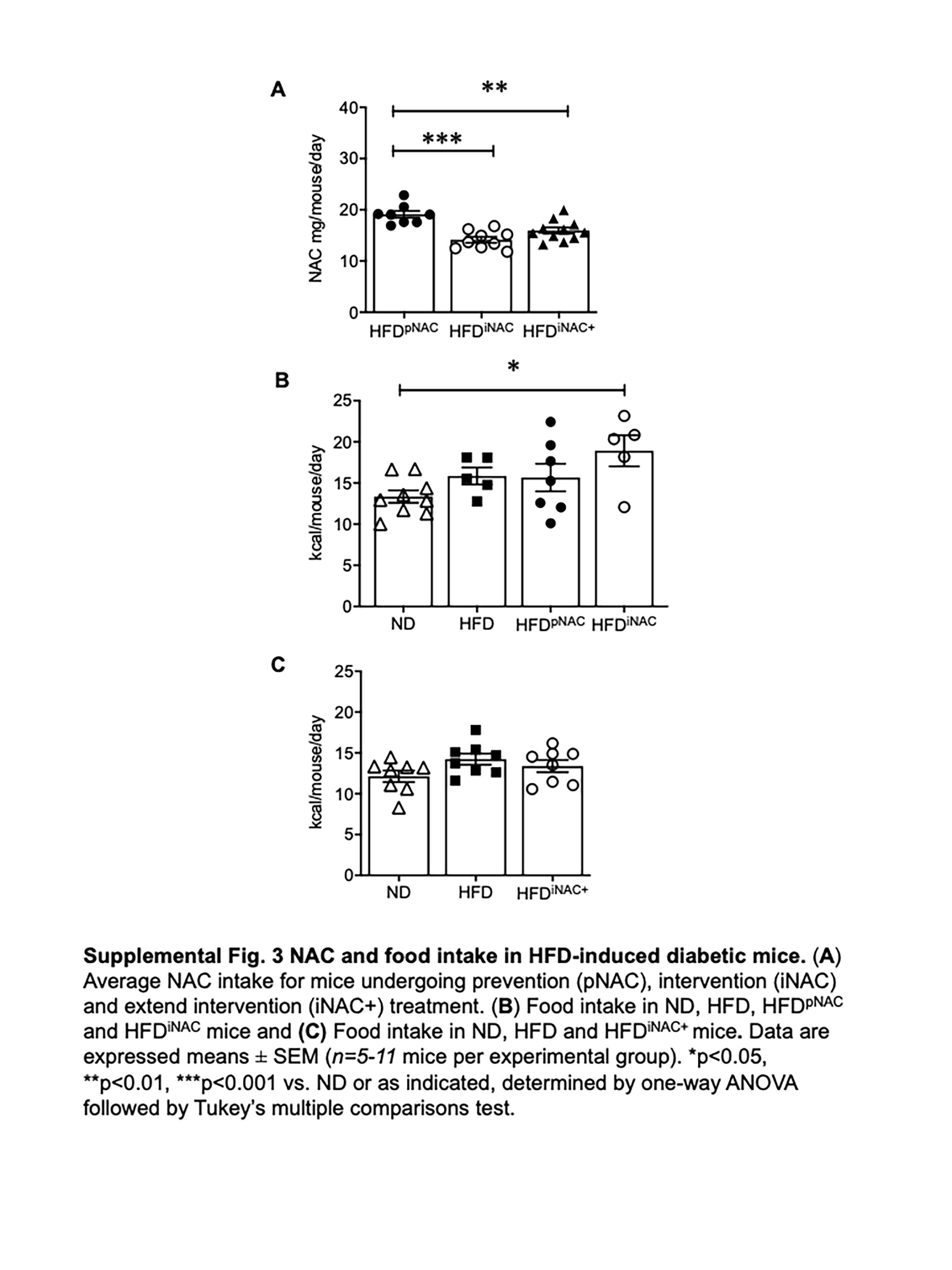

Supplement: Supplementary Figure 3 — NAC and food intake in HFD-induced diabetic mice. (A) Average NAC intake for mice undergoing prevention (pNAC), intervention (iNAC) and extend intervention (iNAC+) treatment. (B) Food intake in ND, HFD, HFDpNAC and HFDiNAC mice and (C) Food intake in ND, HFD and HFDiNAC+ mice. Data are expressed means ± SEM (n=5-11 mice per experimental group). *p<0.05, **p<0.01, ***p<0.001 vs. ND or as indicated, determined by one-way ANOVA followed by Tukey’s multiple comparisons test. [file Image_3.tif]
