# Supplementary material for: N-acetyl-L-cysteine treatment reduces beta-cell oxidative stress and pancreatic stellate cell activity in a high fat diet-induced diabetic mouse model
Source: Front Endocrinol (Lausanne). 2022 Aug 25;13:938680. doi: 10.3389/fendo.2022.938680 (PMC9452715; doi:10.3389/fendo.2022.938680)
Supplement: Supplementary file 6 [file Table_1.docx]

**SUPPLEMENTARY TABLES**

**Supplemental Table 1: Antibodies for immunohistochemistry/immunofluorescence**

| **Primary Antibody** | | | **Source & Isotype** | **Dilution** | **Company** |
| --- | --- | --- | --- | --- | --- |
| Smooth Muscle Actin (α-SMA) | | mouse monoclonal IgG | | 1:200^b^ | Abcam Inc, Cambridge, MA, USA |
| Desmin | rabbit polyclonal IgG | | | 1:200^a^ | Abcam Inc, Cambridge, MA, USA |
| Glucagon | | rabbit monoclonal IgG1 | | 1:100 | Sigma, Saint Louis, Missouri, USA |
| Insulin | | mouse monoclonal IgG1 | | 1:800 | Sigma, Saint Louis, Missouri, USA |
| Insulin | | rabbit polyclonal IgG | | 1:100^a^ | Cell Signaling, Danvers, MA, USA |
| Ki67 | | rabbit monoclonal IgG1 | | 1:200^a^ | Abcam Inc, Cambridge, MA, USA |
| Pdx-1 | | rabbit polyclonal | | 1:800^a^ | Gift - Dr.Wright, University of Vanderbilt, USA |
| 8-OHdG | | mouse monoclonal IgG_2b_ | | 1:50^a^ | Santa Cruz Biotechnology, CA, USA |
|  | |  | |  |  |

^a^ Citrate (pH 6.0) antigen retrieval used; ^b^ 0.2% Triton used
